# Screening Germplasms and Detecting Quantitative Trait Loci for High Sucrose Content in Soybean

**DOI:** 10.3390/plants13192815

**Published:** 2024-10-08

**Authors:** Se-Hee Kang, Seo-Young Shin, Byeong Hee Kang, Sreeparna Chowdhury, Won-Ho Lee, Woon Ji Kim, Jeong-Dong Lee, Sungwoo Lee, Yu-Mi Choi, Bo-Keun Ha

**Affiliations:** 1 Department of Applied Plant Science, Chonnam National University, Gwangju 61186, Republic of Korea; wlsgml7026@naver.com (S.-H.K.); shinsy011123@gmail.com (S.-Y.S.); rkdqudgml555@naver.com (B.H.K.); sreeparna1996@gmail.com (S.C.); dldnjsgh1115@hanmail.net (W.-H.L.); 2 BK21 Interdisciplinary Program in IT-Bio Convergence System, Chonnam National University, Gwangju 61186, Republic of Korea; 3 Advanced Radiation Technology Institute, Korea Atomic Energy Research Institute, Jeongeup 56212, Republic of Korea; dnswl007@naver.com; 4 Department of Applied Biosciences, Kyungpook National University, Daegu 41566, Republic of Korea; jdlee@knu.ac.kr; 5 Department of Crop Science, College of Agriculture and Life Sciences, Chungnam National University, Daejeon 34134, Republic of Korea; sungwoolee@cnu.ac.kr; 6 National Agrobiodiversity Center, National Institute of Agricultural Sciences, RDA, Jeonju 54874, Republic of Korea

**Keywords:** soybean, germplasm, sucrose, glucose oxidase, invertase, quantitative trait locus

## Abstract

Sucrose is a desirable component of processed soybean foods and animal feed, and thus, its content is used as an important characteristic for assessing the quality of soybean seeds. However, few studies have focused on the quantitative trait loci (QTLs) associated with sucrose regulation in soybean seeds. This study aims to measure the sucrose content of 1014 soybean accessions and identify genes related to high sucrose levels using QTL analysis. Colorimetric analysis based on the enzymatic reaction of invertase (INV) and glucose oxidase (GOD) was employed to test the germplasms. A total of six high-sucrose genetic resources (IT186230, IT195321, IT263138, IT263276, IT263286, and IT276521) and two low-sucrose genetic resources (IT025668 and IT274054) were identified. Two F_2:3_ populations, IT186230 × IT025668 and Ilmi × IT186230, were then established from these germplasms. QTL analysis identified four QTLs (*qSUC6.1*, *qSUC11.1*, *qSUC15.1*, and *qSUC17.1*), explaining 7.3–27.6% of the phenotypic variation in the sugar content. Twenty candidate genes were found at the four QTLs. Notably, *Glyma.17G152300*, located in the *qSUC17.1* QTL region, exhibited a 17-fold higher gene expression in the high-sucrose germplasm IT186230 compared to the control germplasm Ilmi, confirming its role as a major gene regulating the sucrose content in soybean. These results may assist in marker-assisted selection for breeding programs that aim to develop soybean lines with a higher sucrose content.

## 1. Introduction

Soybean (*Glycine max* (L.) Merr.) is among the most valuable crops worldwide, providing essential nutrients for both humans and animals. Approximately 352 million tons of soybean are cultivated annually worldwide, with 76% used as livestock feed and 20% as food for humans [1]. In recent years, there has been a notable rise in the demand for meat substitutes and plant-based diets, increasing the popularity of soy-based foods such as tofu, soy milk, and natto. Consequently, the global soy food market has experienced steady growth [2], with an average annual increase of 6.65% [3]. These changes in consumer preferences highlight the need to develop high-quality soybean varieties to ensure food quality.

Soybean seeds are rich in nutrients, containing approximately 40% protein, 20% oil, 35% carbohydrates, and 5% ash [4]. Of the carbohydrate content in soybean, approximately 60% is made up of insoluble sugars, while the remaining 40% is soluble sugars [5]. The soluble sugar fraction consists of 5% sucrose, 1.5% raffinose, 3% stachyose, and trace amounts of fructose and glucose [6]. The presence of sucrose, fructose, and glucose not only enhances the sweetness of soy-based products but also significantly influences their overall quality and taste [7,8,9]. However, oligosaccharides such as raffinose and stachyose, which are classified as raffinose family oligosaccharides (RFOs), cannot be digested by monogastric animals or humans. When metabolized by intestinal bacteria, these oligosaccharides can cause digestive issues such as flatulence and diarrhea, thus affecting their suitability for consumption or use as animal feed [10].

Sucrose strongly influences the sweetness and overall flavor of soy products, including raw soybean, tofu, soy milk, natto, cheonggukjang, and bean sprouts, while also serving as an energy source for the fermentation of products such as natto and cheonggukjang. Additionally, soluble sugars in soybean, such as sucrose and stachyose, are known to promote the growth of bifidobacteria, a beneficial gut bacterium [11]. However, the sucrose content of soybean seeds has also been found to inversely affect the yield of soy milk and tofu, while also affecting the solidity and hardness of tofu [11,12]. Furthermore, high sucrose levels complicate natto processing, unlike stachyose, which is preferred for its beneficial effects [13,14]. In addition to its nutritional advantages, sucrose is the principal product of plant photosynthesis and has a crucial role in the transport process from photosynthetic to non-photosynthetic tissues within the plant. Sucrose also enhances plant resilience against abiotic stress and is important for plant development [15,16].

The sugar content in soybean seeds varies between the stages of seed development and is influenced by environmental conditions during plant growth and seed maturation. During the development and maturation phases, sucrose levels tend to decrease, while indigestible RFOs are present at low levels until three weeks before harvest. At this point, their concentration begins to increase as the seeds approach maturity [17]. An increase in growth temperatures from 21/13 °C to 37/29 °C reduces the sucrose content, whereas the concentrations of stachyose and raffinose slightly decrease [18]. Other studies have also reported that elevated temperatures are associated with lower sucrose levels in seeds [19], with nighttime temperatures having a particularly significant impact on the sugar composition of mature seeds [20,21]. In addition, low-temperature stress can increase sucrose accumulation in the seeds of hydroponically grown soybean plants [22]. These effects of temperature on sucrose levels are associated with genetic factors within the plants [23,24].

Water deficiencies during plant growth reduce the activity of sucrose synthase in soybean, resulting in higher sucrose levels [23,25,26,27]. This is in accordance with reported findings that a lower rainfall, which reduces water availability, is associated with a higher sucrose content [28]. The slow drying of seeds during maturation can also increase their sucrose content up to 5-fold compared to seeds dried under a high relative humidity [29].

Several analytical methods are available to determine the sucrose levels in soybean. For example, high-performance liquid chromatography (HPLC) analysis is highly reliable for the qualitative analysis and quantification of various saccharides [30,31]. However, in large-scale breeding processes where rapid sucrose measurement is required, this method can be impractical due to its high cost and time requirements. Conversely, colorimetric analysis methods using invertase (INV) enzymes that decompose sucrose and enzymes that oxidize glucose (GOD) can save time and money [16,32]. The GOD/INV analysis method converts sucrose into glucose and fructose through invertase and then converts glucose into D-gluconic acid and hydrogen peroxide through GOD. Subsequently, the peroxidase enzyme reacts with the hydrogen peroxide and reduced o-dianisidine to produce oxidized o-dianisidine. The resulting oxidized o-dianisidine exhibits a red color, which can be measured using a spectrophotometer to determine the sucrose content. This method can rapidly analyze large soybean samples that are low in glucose. GOD/INV analysis has a high correlation with HPLC analysis (*r* = 0.91) and is effective for large-scale screening [32].

The sucrose content of soybean is a quantitative trait influenced by multiple genes, meaning that developing varieties with high sucrose levels remains a significant challenge. Candidate genes controlling these traits can be identified through genetic linkage maps and statistical quantitative trait locus (QTL) mapping analysis. Initially, genetic maps were constructed using polymorphic markers such as RFLP, SSR, and RAPD [23]. More recently, however, high-density genetic maps have been constructed using single-nucleotide polymorphism (SNP) markers, which are abundantly and uniformly distributed across the genome, to identify sucrose-related genes [33,34]. Although numerous QTLs associated with proteins and oils have been identified in soybean seeds, research on QTLs for soluble carbohydrates, including sucrose, remains limited. One example is a study that used recombinant inbred lines (RILs) from a cross between ‘Keunolkong’ and ‘Iksan10’ to identify four QTLs linked to sucrose content on chromosomes (Chrs.) 2, 11, and 19 [35]. The QTL on Chr. 19 explained 21.4% of the total phenotypic variation for sucrose content, while the other QTLs accounted for less. In addition, employing RILs derived from a cross between ‘Keunolkong’ and ‘Shinpaldalkong’, two significant QTLs associated with sucrose content were discovered on Chrs. 12 and 16, though they accounted for a relatively low phenotypic variation (<10%) in the sucrose content [36]. More recently, 26 QTLs associated with sugars were identified in a ‘Williams 82’ (F × W82) RIL, with 15 QTLs specifically associated with sucrose distributed across 13 chromosomes (1, 2, 3, 4, 5, 6, 8, 9, 10, 13, 17, 18, and 20) [37]. Nonetheless, these QTLs demonstrated only a modest additive effect. In another study, eight QTLs related to sucrose content were discovered across seven chromosomes (6, 7, 9, 13, 17, 19, and 20), with the QTL on Chr. 19 explaining more than 10% of the phenotypic variation [38].

As such, several QTLs associated with soybean seed sucrose levels have been identified in past research, but validation for various genetic backgrounds and environmental conditions is still required. In line with this, in the present study, 1014 soybean accessions were analyzed and screened to identify those associated with stable and high sucrose levels. The objective was to identify QTLs using two populations produced from selected low- and high-sucrose accessions in order to more clearly understand the genetic underpinnings of soybean sucrose content and to contribute to future breeding programs.

## 2. Results

### 2.1. Analysis of the Sucrose Content of 1014 Soybean Accessions

**Figure 1:**
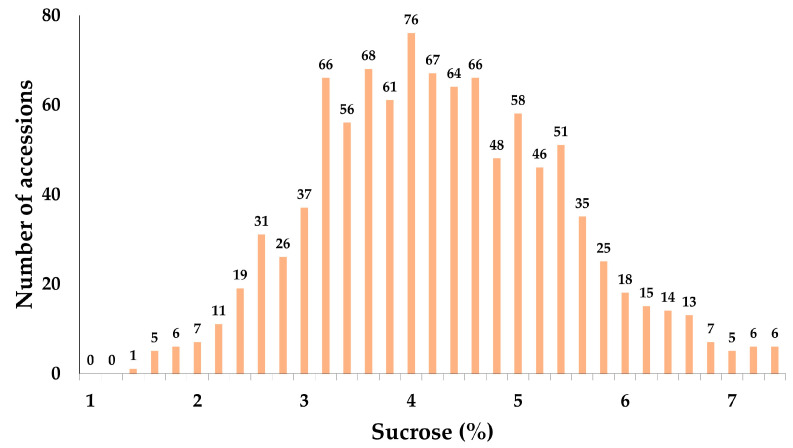
Distribution of sucrose content in 1014 soybean accessions analyzed using GOD/INV method.

GOD/INV analysis was conducted on 1014 soybean accessions, all of which were harvested from a Rural Development Administration (RDA) field in the Republic of Korea in 2019, with the sucrose content ranging from 1.23% to 7.36% and an average of 4.18% (Figure 1). Germplasms with more than 7% or less than 2% sucrose as measured using the GOD/INV approach were planted at Chonnam National University in 2020. The seeds were subsequently harvested and analyzed using the GOD/INV method. Based on the analysis, the high-sucrose germplasms IT186230, IT195321, IT263138, IT263276, IT263286, and IT276521 and the low-sucrose germplasms IT025668 and IT274054 were selected for further analysis (Table 1).

Of the six high-sucrose-containing germplasms, five originated from Korea (IT186230 from Chungcheongbuk-do, IT263286 from Jeollanam-do, and IT263138, IT263276, and IT276521 from Gyeongnam), while IT195321 originated from Colombia. Of the low-sucrose germplasms, IT274054 was bred in Czechoslovakia, while IT025668 is a wild variety from Korea.

Two-year GOD/INV analysis revealed that the sucrose content was higher in 2021 than in 2020. The sucrose content of the low-sucrose germplasms ranged from 1.23% to 2.18%, while that of the high-sucrose germplasms ranged from 6.34% to 7.77%. High-sucrose IT186230 and low-sucrose IT025668 exhibited the most consistent sucrose content, with the levels for IT186230 being higher than those of Ilmi, a high-sucrose control variety.

### 2.2. Sucrose Content Analysis of Two F_2:3_ Populations

**Figure 2:**
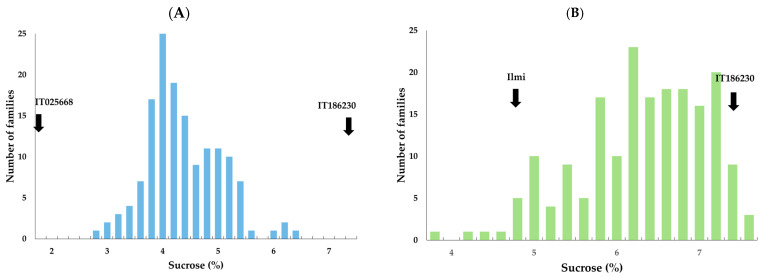
Frequency distribution of sucrose content in F_2:3_ populations derived from crosses of (**A**) IT186230 × IT025668 and (**B**) Ilmi × IT186230.

IT186230, which has the highest sucrose content, IT025668, which had the lowest, and the elite cultivar Ilmi were used as parents to develop two mapping populations. The two F_2:3_ families, IT186230 × IT025668 and Ilmi × IT186230, consisted of 147 and 188 lines, respectively. The sucrose content of the two families was evaluated using GOD/INV analysis (Figure 2).

The sucrose content of the parental lines Ilmi, IT186230, and IT025668 was 4.73%, 7.25%, and 1.73%, respectively (Table 2), whereas that of the IT186230 × IT025668 F_2:3_ family ranged from 2.66% to 6.22%, with an average of 4.27% and a coefficient of variation of 15.33. This family had a right-tailed distribution, and the heritability was 0.96. The sucrose content of the Ilmi × IT186230 F_2:3_ family ranged from 3.79% to 7.59%, with a mean of 3.79% and a coefficient of variation of 12.38. This family had an extended left tail, and the progeny exhibited transgressive segregation that exceeded the sucrose content of the parent lines and a high heritability of 0.95. This suggests that the crossbred soybean variety may have a higher sucrose content, indicating a cumulative effect for this trait.

### 2.3. Genotyping and Linkage Map Construction

Genotyping of the IT186230 × IT025668 F_2_ plants revealed a total of 2710 polymorphic SNP markers between the two parents, of which 1938 were successfully mapped to 20 soybean chromosomes (Supplementary Table S1A and Figure S1A). The genetic map spanned a cumulative distance of 2954.5 cM, and the average distance between adjacent SNPs was 1.5 cM. Each linkage group ranged from 100.7 cM (Chr. 16) to 192.6 cM (Chr. 6). The fewest number of SNP markers was 65 for Chr. 1, while the highest number was 138 for Chr. 2.

In the Ilmi × IT186230 170 F_2_ plants, a total of 1651 SNP markers from among 2469 polymorphic SNPs between the two parents were successfully mapped to 20 soybean chromosomes (Supplementary Table S1B and Figure S1B). The genetic map spanned a cumulative distance of 2754.9 cM, with an average distance of 1.7 cM between adjacent SNPs. The range of distances within the genetic map varied, ranging from 62.2 cM (Chr. 15) to 186.8 cM (Chr. 8). Notably, Chr. 15 had the fewest observed SNP markers (51), while Chr. 13 had the highest (129).

### 2.4. Quantitative Trait Locus (QTL) Analysis

**Figure 3:**
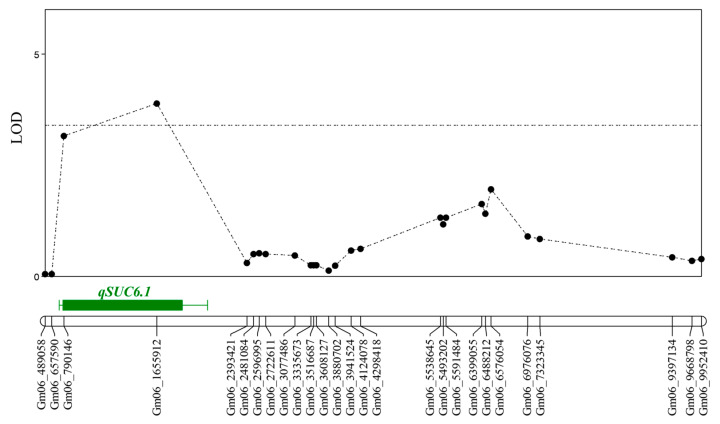
Results of QTL mapping analysis. QTL peak map for soybean Chr. 06 from the F_2:3_ population derived from a cross between IT186230 and IT0255668.

QTLs related to the sucrose content were identified using the logarithm of odds (LOD) approach based on a 1000-permutation test, and genomic regions were identified using interval mapping and the multi-QTL method. In the two populations, a total of four QTLs associated with the sucrose content were identified. The QTL *qSUC6.1* was detected in the F_2:3_ families from the cross IT186230 × IT025668 (Figure 3) and was located between SNP markers Gm06_790146 and Gm06_2393421, explaining 13.4% of the phenotypic variation with an additive effect of 0.39 (Table 3). The LOD for this QTL was 5.12. In the F_2:3_ population, individuals carrying the IT186230 allele had a sucrose content of 4.3%, whereas those carrying the IT025668 allele had a sucrose content of 3.5%.

**Figure 4:**
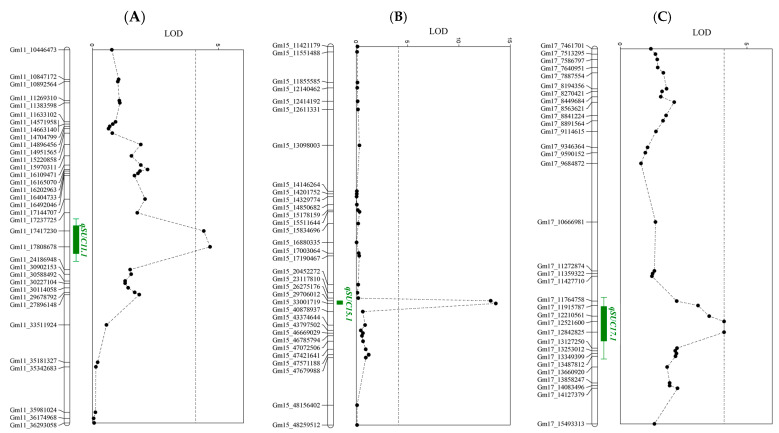
Results of QTL mapping analysis. The QTL peak map for soybean (**A**) Chr. 11, (**B**) Chr. 15, and (**C**) Chr. 17 from the F_2:3_ population derived from a cross between Ilmi and IT186230.

In the Ilmi × IT186230 F_2:3_ family, the QTLs *qSUC11.1*, *qSUC15.1*, *and qSUC17.1* were detected on Chrs. 11, 15, and 17, respectively (Figure 4). *qSUC11.1* was located between SNP markers Gm11_17237725 and Gm11_24186948, with an LOD score of 5.31 and a low additive effect of −0.32 (Table 4). The average sucrose content of lines carrying the IT186230 allele was 6.6%, explaining 9.6% of the total phenotypic variation. *qSUC15.1* was located between SNP markers Gm15_29706012 and Gm15_40878937, explaining 27.6% of the phenotypic variation with the highest LOD score of 13.6. The average sucrose content of the F_2:3_ family with the Ilmi allele was 6.8%, while the line with the IT186230 allele had a sucrose content of 5.7%. *qSUC17.1* was located between SNP markers Gm17_12210561 and Gm17_13127250, representing only 7.3% of the phenotypic variation, with the lowest LOD value of 4.1. The F_2:3_ family with the IT186230 allele had a sucrose content of 6.4%.

### 2.5. Identification of Candidate Genes for Sucrose Content

Using the Wm82.a2.v1 reference genome for soybean, 20 candidate genes associated with the sucrose content were discovered across four QTL regions (Table 5). According to their functions, these 20 genes were classified into five categories. In the energy metabolism series, *Glyma.06G015900* and *Glyma.11G148900* contribute to glucose and glycerol metabolism as dehydrogenases, while *Glyma.06G028800* regulates glucose metabolism by decomposing fructose-2,6-bisphosphate. The starch synthesis pathway involves four family genes related to carbohydrate storage: *Glyma.06G011700*, *Glyma.06G018000*, *Glyma.06G030400*, and *Glyma.06G032500*. In the sugar transporter group, *Glyma.06G015000* and *Glyma.17G152300* are responsible for the transport of sugar molecules, and *Glyma.17G137200* and *Glyma.17G152400* are responsible for nucleotide-sugar transport. Additionally, *Glyma.15G210400* and *Glyma.15G211800* are SWEET proteins that act as sugar efflux transporters. For the glycosylation and sugar modification group, *Glyma.11G150600* catalyzes the conversion of sugar-1-phosphate into GDP-sugar, while *Glyma.15G221300* and *Glyma.17G137500* catalyze the glycosylation reaction by adding glucose or galactose. Finally, for other functions, *Glyma.15G210200* performs protein phosphorylation, and *Glyma.15G210100*, *Glyma.15G212600*, and *Glyma.17G138700* are involved in trehalose-6-phosphate synthesis and glutathione S-transferase for stress protection.

### 2.6. mRNA Expression Analysis of Sucrose-Related Genes

**Figure 5:**
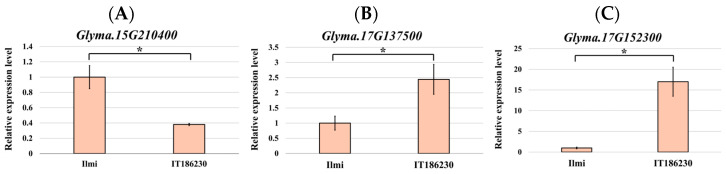
Relative expression levels of three candidate genes in seeds of IT186230 and Ilmi during the intermediate stage between R5 and R6 in seed development. (**A**) *Glyma.15G210400*. (**B**) *Glyma.17G137500*. (**C**) *Glyma.17G152300*. *GmActin11* was used as an internal control. Results are expressed as the mean and standard error (SE). * Indicates a significant difference in relative expression levels between IT186230 and Ilmi at the 0.05 level, determined using Student’s *t*-tests.

To analyze the mRNA levels of the identified sucrose-related genes in soybean seeds, qRT-PCR was employed to quantify the expression levels at the intermediate stage between R5 and R6 of seed development in both IT186230 and Ilmi (Supplementary Table S2 and Figure 5). Significant differences in the expression of *Glyma.15G210400*, *Glyma.17G137500*, and *Glyma.17G152300* were observed (Figure 5). The expression of *Glyma.15G210400* was 62% higher in Ilmi than in IT186230. In addition, the expression levels of *Glyma.17G137500* and *Glyma.17G152300* in IT186230 were 2.44-fold and 17-fold higher than IT186230 in Ilmi, respectively.

## 3. Discussion

Sucrose, the main water-soluble sugar in soybeans, greatly influences the taste and quality of seeds and is easily digestible by humans and monogastric animals, enhancing nutritional efficiency [23]. A high sucrose content improves flavor and consumer acceptance, making it desirable for food-grade soybeans. However, research on sucrose levels has been limited compared to studies on protein and oil content. Sucrose accumulation is controlled by multiple quantitative genes and is influenced by genotype and environmental interactions. The sucrose-related QTLs identified in this study could aid breeding programs to enhance soybean quality through marker-assisted selection

HPLC is widely used for the quantitative and qualitative analyses of sucrose, but it is expensive and time-consuming [16,23,30]. To address these issues, this study employed cost-effective and time-efficient GOD/INV analysis to measure the sucrose content of 1014 accessions (Figure 1). GOD/INV analysis proved to be very effective for accessions, and experimental results demonstrated that it is a viable alternative for the rapid and economical assessment of sucrose content (Table 1).

In the present study, the sucrose content varied between 1.71% and 7.77% among 1014 soybean accessions collected from various countries, including Korea. Notably, five of the high-sucrose germplasms and one low-sucrose germplasm originated from Korea. The selected high-sucrose, low-sucrose, and control accessions showed higher sucrose content in 2021 compared to 2020. Although differences in other environmental conditions were minimal, the total monthly precipitation in 2020 was nearly double that of 2021, indicating that 2021 was drier. This is consistent with findings that sucrose content tends to increase in dry conditions (Supplementary Table S3) [39]. From the accessions, IT186230, which had the highest sucrose content, and IT025668, which had the lowest, were selected as parent lines for QTL mapping analysis. Additionally, Ilmi, a known high-sucrose control variety, was also chosen as a parent line. Two F_2:3_ populations were produced to explore QTLs related to sucrose content. We identified four significant QTLs (*qSUC6.1*, *qSUC11.1*, *qSUC15.1*, *and qSUC17.1*) related to sucrose in the two populations derived from the three parental combinations. In the IT186230 × IT025668 F_2:3_ family, *qSUC6.1* was closely linked to the SNP marker Gm06_1655912, with the allele from IT186230 contributing positively to sucrose content (Table 4). Two sucrose QTLs on Chr. 6 have previously been identified, with one flanked by Satt100 (28.00–41.05 Mb) and another designated as *qSU0601* (43.70–44.39 Mb) [36,37]. However, these QTLs were located far downstream from the *qSUC6.1* discovered in this study.

In the Ilmi × IT186230 F_2:3_ family, three QTLs (*qSUC11.1*, *qSUC15.1*, *and qSUC17.1*) were detected on Chrs. 11, 15, and 17, respectively (Table 4). Each QTL was closely linked with SNP markers Gm11_17808678, Gm15_33001719, and Gm17_12842825, explaining 9.6%, 27.6%, and 7.3% of the phenotypic variation in the sucrose content, respectively (Table 4). *qSUC15.1* had a positive additive effect of 0.56, indicating that the allele from Ilmi increased the sucrose content. Conversely, *qSUC11.1* and *qSUC17.1* had a negative additive effect of −0.32 and −0.18, respectively, suggesting that alleles from IT186230 increased the sucrose content. Previous studies have also identified numerous QTLs associated with sucrose content on Chrs. 11, 15, and 17. For example, the presence of QTLs at Satt197 (8.42–9.29 Mb) and *Suc_UA1* (3.84–3.91 Mb) on Chr. 11 has been reported in previous studies [35,40]. Similarly, QTLs on Chr. 15 have been reported on Satt483 (13.41–17.36 Mb) [36]. Recent studies have identified QTLs at *qSUC-10* (4.86 Mb) and *qSU1701* (5.76 Mb) on Chr. 17 [37,38]. Therefore, the position of our QTLs did not overlap with previously reported QTLs, suggesting that they may represent novel QTLs for sucrose content.

Twenty candidate genes related to sucrose were identified at the four QTLs. These genes were divided into five categories: energy metabolism, starch synthesis, sugar transporters, glycosylation and sugar modification, and others. RT-qPCR was performed to evaluate the effect of these candidate genes on sucrose content. Of the candidate genes, *Glyma.15G210400*, *Glyma.17G137500*, and *Glyma.17G152300* were identified as significant contributors to the sucrose content (Figure 5). *Glyma.15G210400* exhibited 62% higher expression levels in Ilmi than IT186230. This gene encodes the sugar efflux transporter protein SWEET41, which controls and facilitates sucrose transport [41]. The SWEET protein is expressed in presumptive phloem parenchyma cells and is a key component of sucrose transport in leaves [42], acting as a uniporter to facilitate the diffusion of sugars across the cell membrane and mediating sucrose efflux from the presumptive phloem parenchyma to the phloem apoplasm [43], and its homolog AtSWEET2 is involved in regulating sucrose accumulation in roots by controlling the transfer of sucrose from the cytosol to the vacuole in Arabidopsis [44,45]. This regulation plays a crucial role in sucrose storage and distribution within the plant cells. For example, in *Malus domestica*, the SWEET gene, along with other sugar transporter genes, was involved in sugar accumulation, and sugar content was associated with the SWEET gene [46]. Therefore, the SWEET41 protein likely plays a significant role in enhancing sucrose levels in soybean.

The expression levels of *Glyma.17G137500*, which encodes a uridine diphosphate-dependent glycosyltransferase (UGT), were 2.44-fold higher in IT186230 than in Ilmi. UGTs catalyze the transfer of sugars to various substrates using UDP as a sugar donor [21] and are involved in plant growth, hormone regulation, and responses to biotic and abiotic stresses [47,48,49,50,51,52]. The glycosylation activity of UGTs affects the solubility and transport of metabolites, including sugars, by forming glycosidic bonds that produce oligosaccharides, polysaccharides, complex sugars (e.g., glycoproteins and glycolipids), and glycoside compounds [53,54]

The expression levels of *Glyma.17G152300* were 17-fold higher in IT186230 compared to Ilmi. This gene was located 1.4 Mbp from *Glyma.17G138500*, which has been reported to be involved in sucrose metabolism [55]. *Glyma.17G152300* functions as a triose-phosphate transporter (TPT), with the phosphate translocator being a key component in photosynthetic sucrose synthesis [56]. This process promotes the storage and utilization of sugars in plant cells, potentially increasing the overall sugar content. Triose phosphates produced during photosynthesis are either exported from the chloroplasts or temporarily stored as starch within the chloroplasts [35,57,58]. Additionally, triose phosphates released into the cytoplasm are converted into sucrose. This suggests that *Glyma.17G152300* may play a significant role in regulating the sucrose content.

## 4. Materials and Methods

### 4.1. Plant Materials

In the present study, 1014 soybean accessions were sourced from the gene bank at the National Agricultural Biodiversity Center of the Rural Development Administration (RDA), Republic of Korea. These germplasms were sourced from 74 countries, including Korea (454), China (117), the United States (104), and Japan (49), with 42 having an unknown origin. Seeds from each accession were sown in an RDA experimental field (35°49′53″ N, 127°03′50″ E) on June 3 and 4, 2019, with 18 seeds from each accession planted in 0.9 m × 0.15 m plots. To maintain the purity of these lines, seeds were multiplied under controlled conditions, and three individual plants were harvested. The harvested seeds were then used for sucrose content analysis. Ilmi, known for having the highest sucrose content among cultivated varieties in Korea, served as the control [32]. The control and germplasms with a sucrose content of more than 7.5% or less than 2.5% were selected and sown at the Chonnam National University experimental site (35°10′32″ N, 126°54′25″ E) in June 2020 and 2021 to validate the sucrose content.

### 4.2. GOD/INV Analysis of the Sucrose Content

In this process, seed powder, prepared by grinding seeds from different individual plants separately, was passed through a 500 μm mesh sieve, dried at 105 °C for 1 h, and then used for analysis [32]. In this process, seed powder was passed through a 500 μm mesh sieve, dried at 105 °C for 1 h, and then used for analysis. The dry seed powder was mixed with distilled water (DW) in a 10:1 (*v*/*w*) ratio, vortexed to achieve homogeneity, and extracted at 50 °C for 2 h at 200 rpm. To purify the sucrose extract, the mixture was centrifuged at 11,300 rpm for 10 min, and the supernatant was extracted. This process was repeated twice.

For the assay, 5 μL of the extracted sucrose sample was placed into a 96-well culture plate, to which 85 μL of deionized water and 10 μL of INV were added. This mixture was then incubated at 55 °C for 10 min. Following this, 200 μL of GOD reagent was added to each well, and the plate was incubated at 50 °C for 20 min, after which it was left to cool at room temperature for 5 min. The absorbance at 490 nm was measured using an Epoch™ microplate reader (BioTek Instruments, Inc., Winooski, VT, USA). The sucrose content was determined using a standard curve (0%, 0.15%, 0.25%, 0.5%, 0.75%, and 1%). Each sample was analyzed in duplicate for biological replicates and in triplicate for technical replicates.

### 4.3. Population Development

IT186230 and IT025668 were selected for their high and low sucrose content, respectively. Ilmi, a Korean cultivated soybean genetic resource with a high sucrose content, was also used as a parent line. Two F_1_ plants, derived from the crosses IT186230 × IT025668 and Ilmi × IT186230, were produced at Chonnam National University during the summer of 2020. The F_1_ plants were grown in a greenhouse to produce F_2_ seeds over the winter of 2020–2021. In the summer of 2021, F_2_ seeds were sown at 20 cm intervals in a plot at Chonnam National University. Following this, a total of 147 individual F_2:3_ lines were produced from the IT186230 × IT025668 family, while 188 individual F_2:3_ lines were grown from the Ilmi × IT186230 family. The sucrose content of the F_2:3_ seeds was then determined using the GOD/INV method.

### 4.4. DNA Extraction and SNP Genotyping

In 2021, fresh leaves from parent and F_2_ plants were collected, and genomic DNA was extracted using the modified cetyltrimethylammonium bromide (CTAB) method [59]. A Nanodrop ND-2000 spectrophotometer (Thermo Fisher Scientific, Waltham, MA, USA) was used to assess the quality and concentration of the genomic DNA. SNP genotyping of the two F_2_ populations along with the parental plants (IT186230, IT025668, and Ilmi) was carried out by TNT Research Company in Anyang, South Korea, employing a Soy SNP 6K Illumina Bead Chip (Illumina, San Diego, CA, USA). SNP alleles were determined using Genome Studio version 2.0 software (Illumina).

### 4.5. Genetic Mapping and QTL Analysis

SNP markers that demonstrated polymorphisms between parents were selected from the Soy SNP 6K array genotyping data. To construct a genetic linkage map for the F_2_ populations, the Kosambi mapping function in Joinmap v4.1 (Kyazma, Wageningen, The Netherlands) was utilized. QTL analysis was carried out using interval mapping and the multi-QTL method in MapQTL 6.0 (Kyazma, Wageningen, The Netherlands). A 1000-permutation test was conducted to establish the LOD significance threshold [60]. Location maps and LOD graphs of the QTLs were generated using MapChart 2.2. The search for candidate genes used genomic data from Wm82.a2.v1 and the identified QTL locations for sucrose content. SoyBase (www.soybase.org) provided the platform for exploring these QTL regions and accessing gene annotations from the soybean reference genome.

### 4.6. RT-qPCR Analysis

RNA was extracted from the intermediate stage of R5 and R6 from the parental lines Ilmi and IT186230 using an RNeasy Plant Mini Kit (Qiagen, Hilden, Germany). The purity and concentration of the extracted RNA were assessed using a Nano-MD spectrophotometer (Thermo Scientific, Waltham, MA, USA) and electrophoresis with 1.5% agarose gel. The RNA concentration was adjusted to 2.5 ng/µL, and complementary DNA (cDNA) synthesis was subsequently conducted with a Super Script™ III First-Strand Synthesis SuperMix kit (Invitrogen, Carlsbad, CA, USA). Quantitative PCR (qPCR) analysis was conducted on an ABI Step On ePlus system (Applied Biosystems, Waltham, MA, USA) using iQ™ SYBR Green SuperMix (Bio-RAD, Hercules, CA, USA) for detection. The reaction mixture (20 μL) consisted of 2 μL of cDNA (50 ng/μL), 10 μL of 2× SYBR Green Supermix, 3 μL of gene-specific RT-qPCR primers, and 5 μL of DW. The RT-qPCR conditions included an initial denaturation at 95 °C for 3 min on a 96-well optical reaction plate (Applied Biosystems), followed by 45 amplification cycles. Each cycle consisted of 15 s of denaturation at 95 °C and a 60 s annealing/extension phase at 60 °C. The 2^−ΔΔCT^ method was used to determine the relative expression levels of the target genes [18], with Ct values normalized to *GmActin11* as the reference housekeeping gene.

### 4.7. Statistical Analysis

Heritability was estimated using a previously reported method and calculated using the following equation [61]:

$$h^{2}=\frac{\sigma_{G}^{2}}{\sigma_{G}^{2}+\sigma_{E}^{2}}$$

For statistical analysis, IBM SPSS Statistics 27 (IBM, Armonk, NY, USA) served as the primary tool. The influence of genotype variation on gene expression was determined by applying Student’s *t*-tests. Results with statistical significance are marked with an asterisk (*) for *p* < 0.05 and with double asterisks (**) for *p* < 0.01. Instances where differences were not statistically significant are labeled as *ns*.

## 5. Conclusions

In the present study, we analyzed the sucrose content of 1014 soybean accessions using the GOD/INV method to select high-sucrose varieties. As a result, the high-sucrose germplasm IT186230 and the low-sucrose germplasm IT025668 were selected, and two mapping populations were established using the high-sucrose control Ilmi.

QTL analysis of these two populations identified four QTLs: *qSUC6.1*, *qSUC11.1*, *qSUC15.1*, and *qSUC17.1.* Twenty candidate genes associated with the sucrose content were identified within these four QTL regions. Of these, three genes—*Glyma.15G210400*, *Glyma.17G137500*, and *Glyma.17G152300*—exhibited significant differences in their expression levels between IT186230 and Ilmi. Notably, *Glyma.17G152300* exhibited 17-fold higher expression levels in IT186230 than in Ilmi, confirming its role as a major determinant of sucrose content.

These results suggest that IT186230 is suitable for the development of high-sugar soybean varieties, thus expanding knowledge of the genetic basis for soybean sucrose levels and potentially assisting in the development of breeding programs for food-grade soybean varieties.

## Figures and Tables

**Table 1:** Sucrose content for eight soybean accessions grown over two years (2020 and 2021). The results are expressed as the mean and standard deviation (SD).

	Genotype	Origin	Sucrose Content (%)	
			2020	2021
High sucrose	IT186230	KOR	6.44 ± 0.09	7.77 ± 0.03
	IT195321	COL	6.34 ± 0.06	6.60 ± 0.07
	IT263138	KOR	7.37 ± 0.18	7.61 ± 0.01
	IT263276	KOR	6.97 ± 0.04	7.43 ± 0.09
	IT263286	KOR	7.27 ± 0.25	7.65 ± 0.00
	IT276521	KOR	6.60 ± 0.37	7.49 ± 0.06
Low sucrose	IT025668	KOR	1.23 ± 0.00	1.71 ± 0.03
	IT274054	CSK ^†^	1.85 ± 0.11	2.18 ± 0.05
Control	Ilmi	KOR	-	7.39 ± 0.03

^†^ CSK: Czechoslovakia. It separated into the Czech Republic and Slovakia in 1993.

**Table 2:** Descriptive statistics for the sucrose content (%) of parental lines and F_2:3_ populations derived from IT186230 × IT025668 and Ilmi × IT186230.

Populations	Parent		F_2:3_							
	P1 ^†^	P2	N	Min.	Max.	Mean.	CV	Skew.	Kurt.	h^2^
IT186230 × IT025668	7.25	1.73	147	2.66	6.22	4.27	15.33	0.51	0.32	0.96
Ilmi × IT186230	4.73	7.25	188	3.79	7.59	6.21	12.39	−0.60	−0.14	0.95

^†^P1, maternal parent; P2, paternal parent; N, size of population; Min, minimum; Max, maximum; CV, coefficient of variation; Skew, skewness; Kurt, kurtosis; and CV: coefficient of variation.

**Table 3:** Identified QTL related to the sucrose content in the F_2:3_ IT186230 × IT025668 population.

QTL	Chr.	Interval	Physical Interval (Mb)	LOD	Peak or Flanking Marker	IT186230 Allele Effect (%)	IT025668 Allele Effect (%)	R^2^	Additive Effect
*qSUC6.1*	6	Gm06_790146–Gm06_2393421	0.7–2.3	5.12	Gm06_1655912	4.3	3.5	13.4	0.39

**Table 4:** Identified QTLs related to the sucrose content in the F_2:3_ Ilmi × IT186230 population.

QTL	Chr.	Interval	Physical Interval (Mb)	LOD	Peak or Flanking Marker	Ilmi Allele Effect (%)	IT186230 Allele Effect (%)	R^2^	Additive Effect
*qSUC11.1*	11	Gm11_17237725–Gm11_24186948	17.2–24.1	5.31	Gm11_17808678	5.9	6.6	9.6	–0.32
*qSUC15.1*	15	Gm15_29706012–Gm15_40878937	29.7–40.8	13.6	Gm15_33001719	6.8	5.7	27.6	0.56
*qSUC17.1*	17	Gm17_12210561–Gm17_13127250	12.2–13.1	4.1	Gm17_12842825	6.1	6.4	7.3	–0.18

**Table 5:** Candidate genes for seed sucrose content identified in the reference genome based on QTL-related SNPs in the IT186230 × IT025668 (A) and Ilmi × IT186230 (B) mapping populations.

QTL	Gene ID	Physical Position (bp)	Annotation	Protein ID
*qSUC6.1*				
	*Glyma.06G011700*	Gm06:873613..878088	Glucose-1-phosphate adenylyl transferase family protein	
	*Glyma.06G015000*	Gm06:1127434..1132895	Tonoplast monosaccharide transporter 2	
	*Glyma.06G015900*	Gm06:1187405..1190649	Glyceraldehyde-3-phosphate dehydrogenase B subunit	
	*Glyma.06G018000*	Gm06:1359828..1368182	1,4-alpha-glucan-branching enzyme/starch-branching enzyme II	
	*Glyma.06G028800*	Gm06:2237126..2239413	Fructose-2,6-bisphosphatase	
	*Glyma.06G030400*	Gm06:2390673..2396324	Glucose-1-phosphate adenylyltransferase large subunit 2	
	*Glyma.06G032500*	Gm06:2515199..2519958	Glucose-6-phosphate isomerase	
*qSUC11.1*				
	*Glyma.11G148900*	Gm11:11489777..11495616	Glycerol-3-phosphate dehydrogenase	
	*Glyma.11G150600*	Gm11:11808846..11810660	Sugar-1-phosphate guanyl transferase	
*qSUC15.1*				
	*Glyma.15G210100*	Gm15:32175249..32210061	Trehalose-6-phosphate synthase	
	*Glyma.15g210200*	Gm15:32217111..32218567	Protein kinase superfamily protein	
	*Glyma.15G210400*	Gm15:32262290..32264065	RAG1-activating protein 1	SWEET41
	*Glyma.15G211800*	Gm15:32835023..32838031	RAG1-activating protein 1	SWEET42
	*Glyma.15G212600*	Gm15:33402757..33405141	Glutathione S-transferase THETA 2	
	*Glyma.15G221300*	Gm15:39951868..39954192	UDP-glucosyl transferase 73B3	
*qSUC17.1*				
	*Glyma.17G137200*	Gm17:11073919..11077576	Nucleotide-sugar transporter family protein	
	*Glyma.17G137500*	Gm17:11097469..11100931	UDP-glycosyltransferase superfamily protein	
	*Glyma.17G138700*	Gm17:11248118..11252772	Trehalose-phosphate synthase	
	*Glyma.17G152300*	Gm17:12696012..12697117	Triose-phosphate transporter family	
	*Glyma.17G152400*	Gm17:12704557..12706988	UDP-galactose transporter	

## Data Availability

The original contributions presented in the study are included in the article/Supplementary Materials, further inquiries can be directed to the corresponding authors.

## Supplementary Materials

The following supporting information can be downloaded at: https://www.mdpi.com/article/10.3390/plants13192815/s1, Supplementary Table S1: Genetic linkage map details for the F_2_ population derived from the crosses IT186230 × IT025668 (A) and Ilmi × IT186230 (B), Supplementary Table S2: List of primers utilized in the qRT-PCR analysis, Supplementary Table S3. Monthly temperature, humidity, and precipitation data for Gwangju during the soybean cultivation period (from the sowing month of June to the harvest month of October) from 2020 to 2021. Supplementary Figure S1: High-density linkage map using the F_2_ populations derived from the crosses IT186230 × IT025668 (A) and Ilmi × IT186230 (B).
