# Supplementary material for: Screening Germplasms and Detecting Quantitative Trait Loci for High Sucrose Content in Soybean
Source: Plants (Basel). 2024 Oct 8;13(19):2815. doi: 10.3390/plants13192815 (PMC11478759; doi:10.3390/plants13192815)
Supplement: Supplementary file 1 [file plants-13-02815-s001.zip › plants-3205961-supplementary.pdf]

Supplementary Table S1. Genetic linkage map details for the F_2 population derived from the crosses IT186230 × IT025668 (A) and Ilmi × IT186230 (B).

Chr.	(A)			(B)		
	Marker No.	Length (cM)	Average spacing (cM)	Marker No.	Length (cM)	Average spacing (cM)
1	65	147.6	2.3	79	135.1	1.7
2	138	164.9	1.2	92	177.3	1.9
3	81	147.8	1.8	55	134.3	2.4
4	95	139.8	1.5	79	135.3	1.7
5	68	122.1	1.8	71	106.1	1.5
6	111	192.6	1.7	95	163.1	1.7
7	78	166.1	2.1	78	144.8	1.9
8	122	183	1.5	99	186.8	1.9
9	68	139.9	2.1	86	128.6	1.5
10	96	144.5	1.5	73	149.6	2.1
11	95	158.7	1.7	78	147.5	1.9
12	88	125.2	1.4	77	127	1.6
13	129	185.4	1.4	129	165.2	1.3
14	96	133.4	1.4	73	124.1	1.7
15	111	128	1.2	51	62.2	1.2
16	69	100.7	1.5	87	99.7	1.1
17	89	162.3	1.8	81	156.1	1.9
18	132	133.2	1	107	133.5	1.2
19	117	142.9	1.2	86	146.8	1.7
20	90	136.3	1.5	75	131.9	1.8
Total	1938	2954.5		1651	2754.9	
Average	96.9	147.72	1.5	82.55	137.75	1.7

Supplementary Table S2. List of primers utilized in the qRT-PCR analysis.

Gene ID (Glyma.Wm82. a2. v1)	Primer (5′ – 3′)	Product size	Annealing temperature
Glyma.15G210400	F:CATTATCAATGTCCCCCATACCG<br>R: TATAAGGCAACCCGGAGAACA	82 bp	61.7 °C
Glyma.17G137500	F: GGAGCTGCGTTCTGTCCTAT<br>R: TCCTAGTGACGAGTTTGCTGG	119 bp	60.2 °C
Glyma.17G152300	F: CCCAATGCTCGTTGCTTCAG<br>R: CATGCCACAGCAGTCCAAAC	141 bp	59.9 °C
GmActin11	F: GGTGGTTCTATCTTGGCATC<br>R: CTTTCGCTTCAATAACCCTA	82 bp	56.0 °C

Supplementary Table S3. Monthly temperature, humidity, and precipitation data for Gwangju during the soybean cultivation period (from the sowing month of June to the harvest month of October) from 2020 to 2021.

Location	Year	Month	Average temperature (° C)	Average High Temperature (° C)	Average minimum temperature (° C)	Average relative humidity (%)	Monthly combined precipitation (00~24h only) (mm)
Gwangju	2020	6	23.6	28.8	19.6	79	199.9
Gwangju	2020	7	23.4	26.8	20.8	91	533.3
Gwangju	2020	8	27.6	31.6	24.8	89	738.1
Gwangju	2020	9	21.3	25.7	17.9	85	178.3
Gwangju	2020	10	15.7	21.2	11.2	64	12.1
Gwangju	2021	6	23.3	28.3	19.2	76	118.1
Gwangju	2021	7	27	31.4	23.6	82	227.6
Gwangju	2021	8	25.8	30.3	22.5	88	338.7
Gwangju	2021	9	22.9	27.2	19.5	84	131.1
Gwangju	2021	10	17.2	22.7	12.8	77	35.3

* Data obtained from the National Climate Data Center of the Republic of Korea.

A

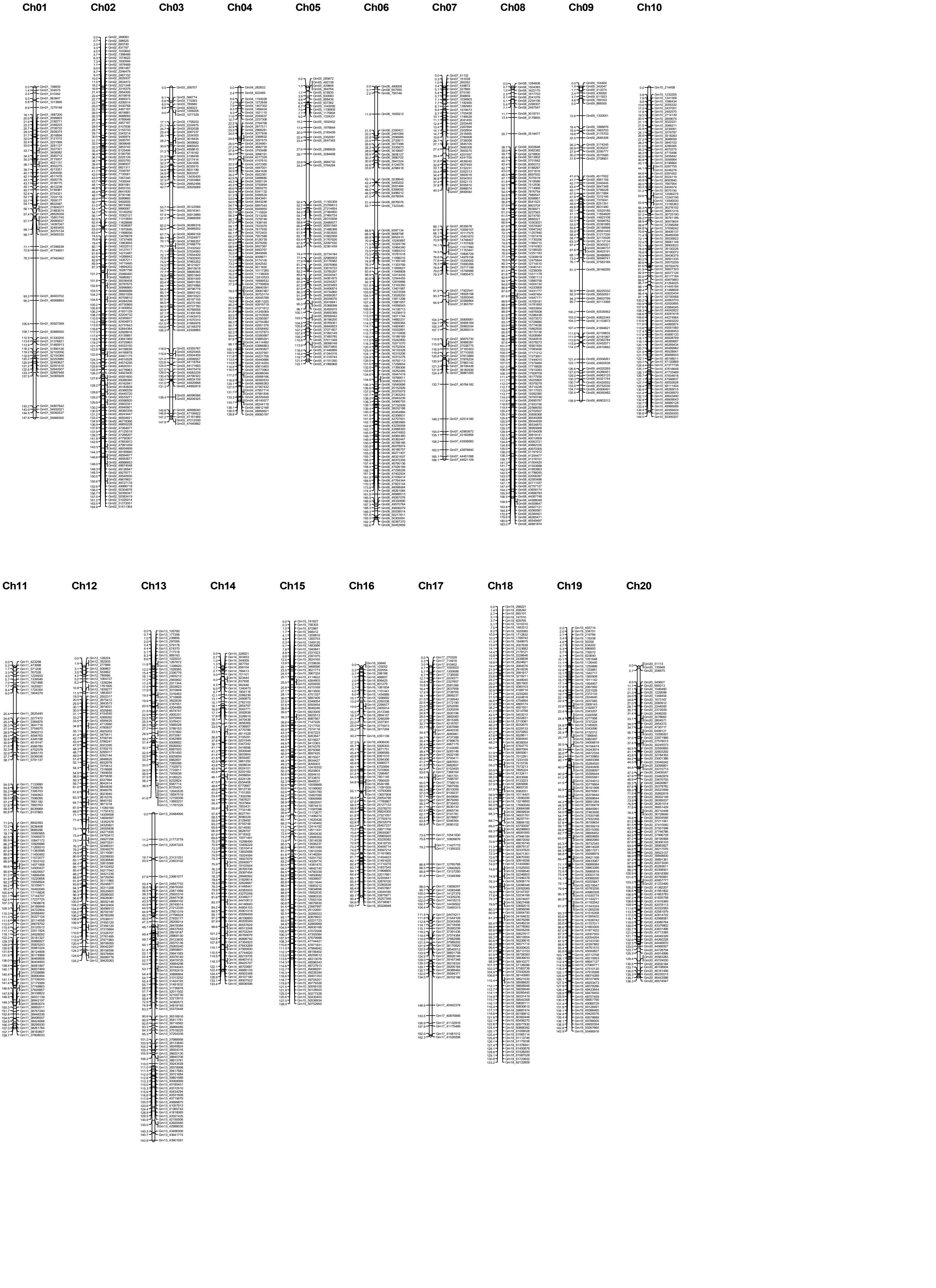
Ch01
Ch02
Ch03
Ch04
Ch05
Ch06
Ch07
Ch08
Ch09
Ch10
Ch11
Ch12
Ch13
Ch14
Ch15
Ch16
Ch17
Ch18
Ch19
Ch20

B

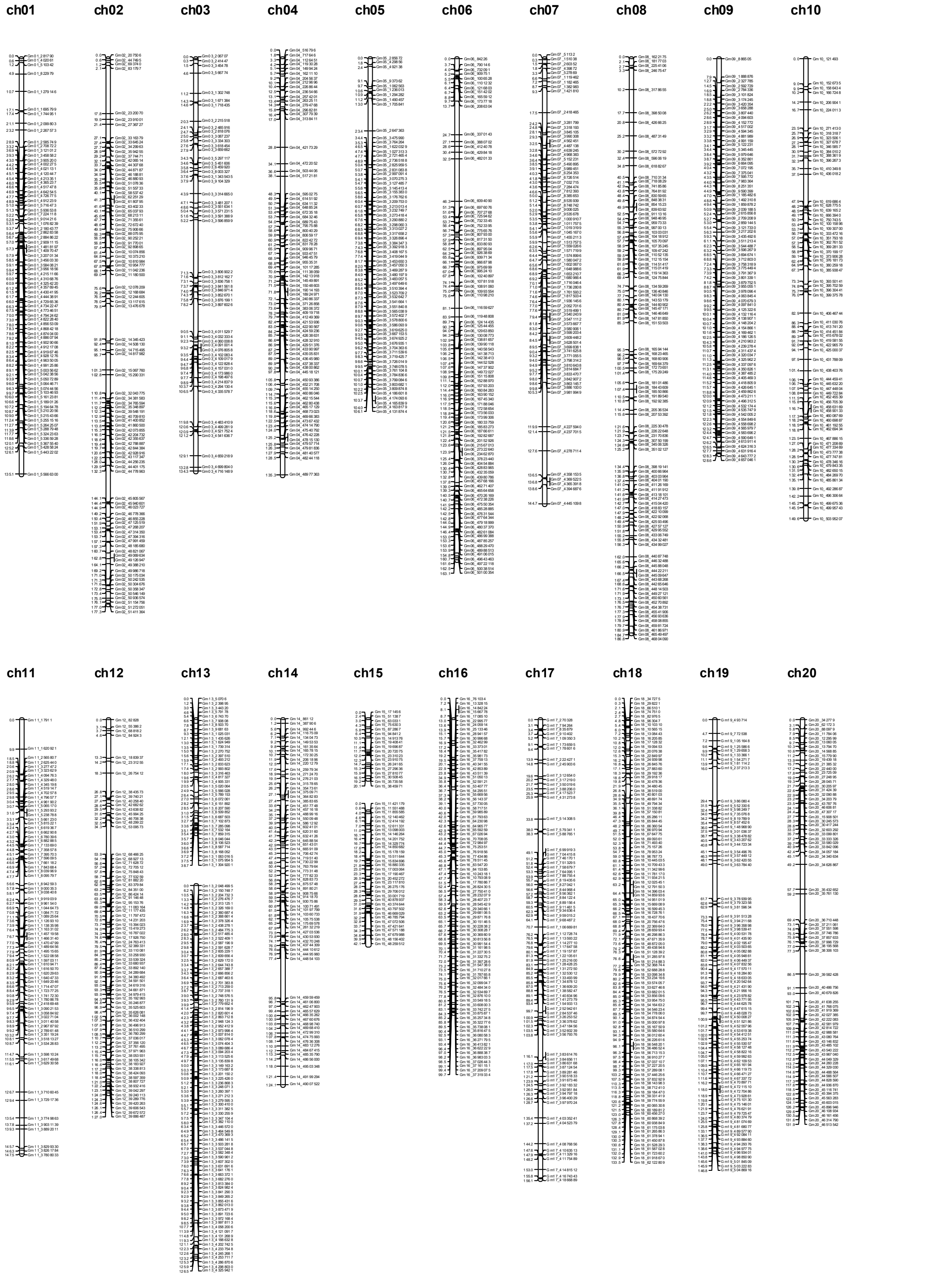

Supplementary Figure S1. High-density linkage map using the F_2 populations derived from the crosses IT186230 × IT025668 (A) and Ilmi × IT186230 (B).
